# Adolescent mothers’ body composition and metabolic hormones (adiponectin, ghrelin, and leptin) in blood and human milk: associations with infant body composition

**DOI:** 10.1038/s41430-026-01784-5

**Published:** 2026-07-21

**Authors:** Verônica Rached, Elaine Pereira de Rezende, Maria Eduarda Leão Diogenes, Marcia Fernanda Taveira, Carmen Marino Donangelo, Flávia Fioruci Bezerra

**Affiliations:** 1 Nutrition Institute, Rio de Janeiro State University, Rio de Janeiro, Brazil; 2 Brazilian National Cancer Institute, Rio de Janeiro, Brazil; 3 School of Nutrition, University of the Republic, Montevideo, Uruguay

**Keywords:** Nutrition, Fat metabolism, Whole body imaging, Paediatrics

## Abstract

**Background/Objectives:**

This study aimed to investigate whether maternal body composition is related to blood and human milk metabolic hormones (adiponectin, ghrelin, and leptin) and whether these hormones are associated with infant body composition and anthropometric indicators.

**Subjects/Methods:**

Fifty-five adolescent mother–infant pairs were evaluated at postpartum. Maternal and infant body composition was assessed using dual-energy X-ray absorptiometry. Leptin, adiponectin, and ghrelin were measured in maternal circulation and human milk. Multiple linear regression models adjusted for days postpartum assessed associations between (1) maternal characteristics and hormones in maternal blood and milk and (2) milk hormones and infant measures (also adjusted for birth weight).

**Results:**

Serum and human milk leptin were directly associated (r = 0.472; *p* = 0.014), whereas serum and human milk ghrelin were inversely correlated (r = −0.320; *p* = 0.017). Greater maternal fat mass, particularly in the arms and android region, was associated with higher leptin concentrations in serum (β = 5.95; 95% CI: 1.65–10.3 and β = 5.90; 95% CI: 0.871–10.9, respectively) and milk (β = 0.294; 95% CI: 0.132-0.455 and β = 0.280; 95% CI: 0.118–0.443, respectively). Higher milk leptin was associated with lower infant length (β = -1.21; 95% CI: −2.24, −0.183). Milk ghrelin was weakly and inversely associated with several infant anthropometric measures, whereas no significant associations were found for milk adiponectin.

**Conclusion:**

The results suggest that maternal leptin in serum and milk, but not ghrelin or adiponectin, is associated with maternal adiposity. Leptin and ghrelin in human milk may be involved in infant growth and body composition, whereas adiponectin does not appear to play a major role.

## Introduction

Metabolic hormones in human milk, including leptin, adiponectin, and ghrelin, have been studied for their potential influence on infant feeding behavior, growth, and body composition [1, 2]. These hormones may originate from synthesis in the mammary gland [3–5], but evidence also suggests that maternal circulating levels contribute to their concentrations in breast milk, possibly through uptake and concentration in mammary tissue [3, 4, 6]. Consequently, maternal factors known to influence circulating hormone levels—such as pre-pregnancy and postpartum body mass index (BMI) and gestational weight gain [7–9]—may also affect hormone concentrations in human milk.

Regardless of their source, experimental studies indicate that metabolic hormones in breast milk can cross the infant’s intestinal mucosa and enter the bloodstream [10, 11]. Human milk concentrations of leptin and adiponectin have been inversely associated with infant growth indicators, including weight, length, and fat mass during the first 2 years of life [12–16]. In contrast, findings regarding ghrelin are inconsistent: while some studies report no association with several growth outcomes [6, 17], others describe either direct [18] or inverse [16] associations with infant weight.

Only a limited number of studies have simultaneously examined maternal blood and human milk concentrations of leptin and adiponectin in relation to mother–infant body composition [15, 19]. Furthermore, many of these studies relied on less accurate methods for assessing body composition, such as skinfold thickness [15, 20, 21] or bioelectrical impedance analysis [19].

We hypothesized that maternal body composition is associated with circulating and human milk concentrations of adiponectin, ghrelin, and leptin, as well as with infant body composition. Therefore, this study aimed to investigate: (1) whether maternal body composition measured by dual-energy X-ray absorptiometry (DXA) is associated with blood and human milk concentrations of these hormones; and (2) whether human milk hormone concentrations at approximately 1 month postpartum are related to infant body composition assessed by DXA and to anthropometric indicators. To address these questions, we conducted a secondary post hoc analysis using data from our previously reported clinical trial (ClinicalTrials.gov: NCT01732328), which investigated the effects of calcium plus vitamin D supplementation during adolescent pregnancy on maternal bone mass changes during 1 year postpartum and on infant bone mass at 1 month after delivery [22–24].

## Materials and methods

### Study design

This study was conducted in a convenience sample from a previously reported longitudinal clinical trial [22–24]. A cross-sectional analysis was conducted with 55 mother–infant pairs at approximately 5 weeks postpartum, when maternal and infant data were collected concurrently. Adolescents aged 13–19 years, primigravidae with singleton pregnancies, gestational age between 23 and 29 weeks, intending to breastfeed, and without chronic diseases or pregnancy complications were recruited during prenatal care at the Federal University of Rio de Janeiro (2009–2011). Written informed consent was obtained from all participants and their parents or legal guardians before enrollment in the study. Detailed description of the study protocol, sample size estimate, ethical approval, and primary results of the trial (ClinicalTrials.gov: NCT01732328) were published [22–24].

For the present analyses, the minimum sample size was estimated using effect sizes previously reported [8] for associations between maternal weight and maternal leptin concentrations in serum and human milk, as well as between human milk leptin and infant length. Calculations were performed in G*Power software for multiple linear regression models, assuming a 95% confidence level and 80% statistical power. The estimated minimum sample size ranged from 15 to 33 participants, depending on the model. As the final sample comprised 55 participants, the study was adequately powered. Additional post hoc analyses indicated statistical power >98% for the associations between (1) maternal fat mass (kg) and serum leptin, (2) maternal fat mass (kg) and human milk leptin, and (3) human milk leptin and infant length.

### Maternal and infant data collection

Maternal age, age at menarche, gestational age, and pre-pregnancy weight were collected at recruitment (~26 weeks of gestation). Data on childbirth, newborn characteristics, gestational weight gain, and breastfeeding practices (frequency, duration, feeding intervals, consumption of other foods) were obtained at ~5 weeks (min-max: 10–58 days) postpartum. On the same day, maternal and infant anthropometry, body composition, and blood and human milk samples were collected. Breastfeeding was classified as exclusive, predominant, or complementary [25].

Maternal blood samples (20 mL) were collected in the morning (~8 a.m.) after an overnight fast, centrifuged, and stored at −80 °C. Immediately before blood sampling, pre-feed morning mature human milk samples (~20 mL) were collected by manual expression of the breast not sucked after the last feeding. This standardized sampling protocol was adopted to minimize potential diurnal variability and within-feed differences in milk composition. The time elapsed since the last feeding was recorded (range: 1–3 h). Whole samples were stored at −80 °C. Leptin was measured in serum, and adiponectin and total ghrelin in plasma. Hormone concentrations in blood and milk were determined by ELISA (Millipore, USA, MO #EZHL-80SK, #EZHADP-61K, and #EZGRT-89K, respectively). Samples were analyzed in duplicate.

Maternal weight and height were measured using a calibrated electronic scale (Filizola®) and stadiometer (Seca®), and BMI was calculated. Postpartum weight retention was defined as the difference between weight at 5 weeks postpartum and pre-pregnancy weight. Total body mass, lean mass, total and regional fat mass were assessed by DXA (iDXA, enCore 2008 version 12.20, GE Healthcare, USA) [22, 26], and visceral adipose tissue was estimated using CoreScan VAT [26].

Infant body weight was measured using a calibrated pediatric digital scale (Filizola; 0–15 kg), and length was measured with a wooden infantometer accurate to 0.1 cm. Weight gain since birth was calculated, and neonatal and 5-week postpartum z-scores were determined using WHO standards [27]. Whole-body composition was assessed by DXA, as previously described [23].

### Statistical analyses

Variable normality was assessed using the Kolmogorov–Smirnov test. Descriptive analyses were performed to characterize the sample, with data expressed as mean ± standard deviation (SD) or median (25th–75th percentiles). Missing data, such as values below the detection limit and outlying concentration values, were handled using multiple imputation, which yields less biased estimates and better preserves statistical power and uncertainty [28, 29]. Estimates from 20 imputed datasets were pooled. The number of imputations was chosen to ensure stable estimates [28]. The percentage of missing values for each variable ranged from 1 to 18%.

T-tests were used to compare hormone concentrations between maternal blood and human milk. Pearson’s correlation was used to evaluate associations between hormone concentrations in blood and human milk, as well as their associations with the postpartum period. Two multiple linear regression models were tested: (1) Maternal characteristics (eg. BMI, gestational weight gain, body composition) as independent variables with maternal blood and milk hormones as dependent variables, adjusted for postpartum period (days) [4, 30]; and (2) Milk hormone concentrations as independent variables with infant characteristics (growth indicators and body composition) as dependent variables, adjusted for postpartum period (days) and birth weight (g) [1]. Maternal BMI, calcium plus vitamin D supplementation during pregnancy, infant sex, and breastfeeding practices were also tested as potential covariates but excluded due to lack of significance [1]. Analyses were conducted using SPSS 20.0 (IBM Corp), with significance set at *P* < 0.05 and 95% confidence intervals (CI).

## Results

### Mother–infant pair characteristics

The age of the adolescent mothers ranged from 14 to 19 years (mean: 17.3 ± 1.3 years), with an average of 5.3 ± 1.9 years elapsing since menarche (Table 1). Seventy-six percent of adolescents began their pregnancy with adequate BMI-for-age (mean BMI: 21.5 ± 3.9 kg/m²) [27]. Mean total gestational weight gain was above the Brazilian recommendations (13.8 ± 6.4 kg) [31] considering pre-pregnancy BMI. The median postpartum weight retention was 4.1 kg (min-max: 1.6–8.2 kg) (Table 1). According to BMI postpartum, 67.3% had an adequate weight for age, while 30.9% were above the recommendations (20% presented as overweight, and 10.9% as obese) [27].

**Table 1:** Characteristics of the adolescent mothers and infant pairs^a^.

Characteristic	*n*	Mean ± SD	Median (percentile 25–percentile 75)
*Mothers*			
Age (years)	55	17.3 ± 1.3	17.6 (16.3–18.3)
Time elapsed since menarche (years)	55	5.3 ± 1.9	5.3 (3.9–6.7)
Pre-pregnancy BMI (kg/m²)^b^	50	21.5 ± 3.9	20.6 (19.1–23.9)
Gestational age at delivery (week)	54	39.3 ± 1.5	39.4 (38.3–40.3)
Gestational weight gain (kg)	47	13.8 ± 6.4	12.0 (9.6–16.2)
Postpartum weight retention (kg)	51	5.1 ± 5.7	4.1 (1.6–8.2)
BMI (kg/m²)^b^	55	23.7 ± 4.3	23.4 (21.0–25.9)
Body composition	55		
Total body mass (kg)		60.2 ± 12.7	58.7 (51.6–66.0)
Total body fat (%)		36.1 ± 5.64	35.6 (31.6–39.8)
Total body fat (kg)		21.4 ± 7.97	20.3 (16.4–25.7)
Lean mass (kg)		38.8 ± 5.64	38.3 (35.2–41.6)
Blood hormones			
Adiponectin (µg/ml)	55	5.85 ± 2.50	5.64 (4.36–6.39)
Ghrelin (pg/ml)	55	649 ± 395	548 (360–779)
Leptin (ng/ml)	47	16.5 ± 10.8	16.1 (5.6–23.8)
Human milk hormones			
Adiponectin (µg/ml)	55	0.33 ± 0.08	0.32 (0.29–0.38)
Ghrelin (pg/ml)	54	151 ± 34	151 (134–163)
Leptin (ng/ml)	54	0.44 ± 0.42	0.37 (0.07–0.62)
Breastfeeding practices	55		
Exclusively, *n* (%)		45 (81.8)	
Predominantly, *n* (%)		7 (12.7)	
Complementary, *n* (%)		3 (5.5)	
*Infants*			
Childbirth	55		
Vaginal, *n* (%)		28 (50.9)	
Sex	55		
Male, *n* (%)		30 (54.5)	
Birth weight (kg)	55	3.24 ± 0.54	3.21 (2.87–3.60)
Birth height (cm)	55	48.5 ± 2.7	49.0 (47.0–50.0)
Birth head circumference (cm)	53	34.3 ± 1.5	34.0 (33.0–35.5)
*z*-score at birth^c^			
Weight-for-age	55	−0.17 ± 1.14	−0.22 (−0.96 to 0.51)
Length-for-age	55	0.55 ± 1.40	−0.47 (−1.52 to 0.19)
BMI-for-age	55	0.18 ± 1.23	0.43 (−0.51 to 0.98)
Weight-for-length	49	0.44 ± 1.40	0.53 (−0.18 to 1.44)
Head circumference-for-age	53	0.09 ± 1.21	0.10 (−1.15 to 0.95)
Age (days)	55	32.7 ± 10.4	31.0 (26.0–39.0)
Weight gain (kg)	55	0.91 ± 0.57	0.84 (0.46–1.34)
*z*-score^c^	55		
Weight-for-age		−0.49 ± 1.27	−0.58 (−1.46 to 0.45)
Length-for-age		−0.50 ± 1.04	−0.32 (−1.17 to 0.19)
Weight-for-length		−0.49 ± 1.27	−0.58 (−1.46 to 0.45)
BMI-for-age		−0.53 ± 1.11	−0.46 (−1.36 to 0.09)
Body composition	55		
Total body mass (kg)		4.61 ± 0.70	4.56 (4.10–5.17)
Total body fat (%)		20.14 ± 3.84	19.9 (17.70–22.60)
Total body fat (kg)		0.97 ± 0.36	0.94 (0.74–1.11)
Lean mass (kg)		3.65 ± 0.50	3.66 (3.22–4.01)

*BMI* body mass index, *n* sample size, *SD* standard deviation.

^a^Raw data.

^b^Classification according to BMI-for-age [27].

^c^Z-scores were determined according to the WHO child growth standards [27].

**Fig. 1:**
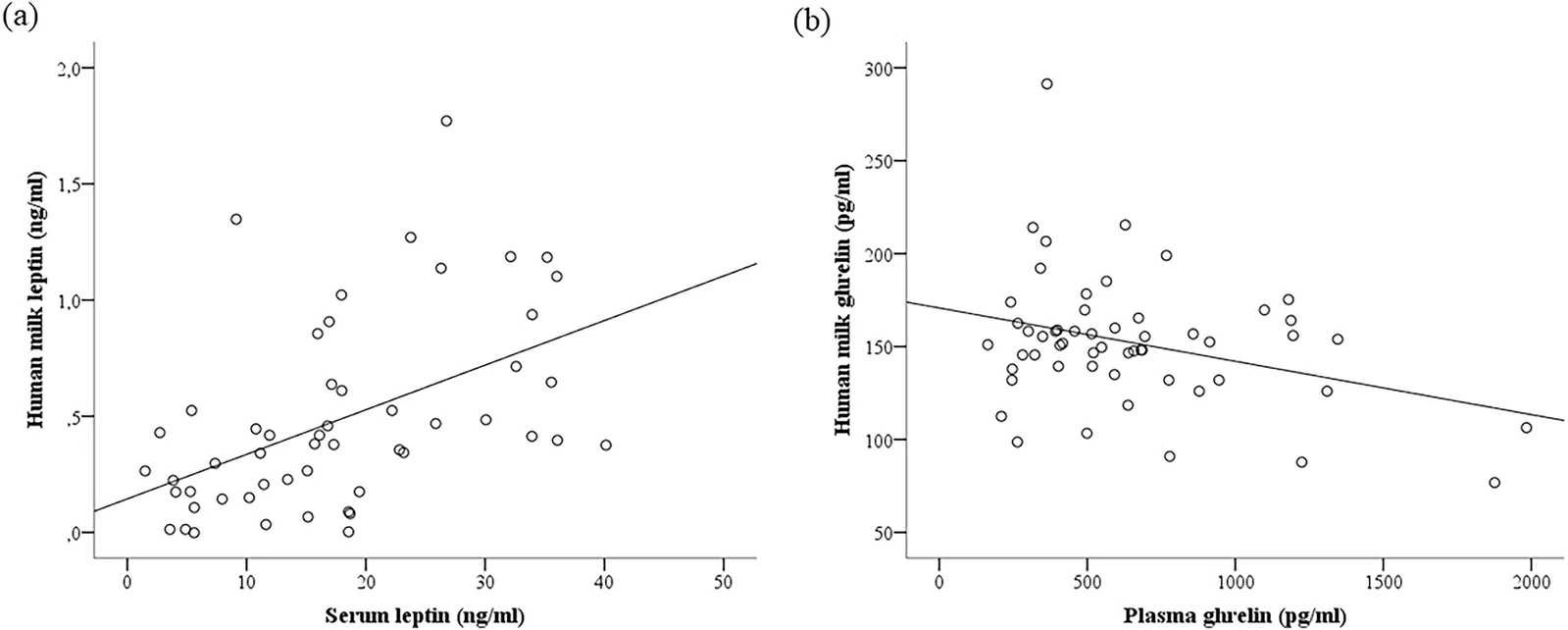
Maternal Blood and Human Milk Hormone Associations. Associations between **a** maternal serum and human milk leptin concentrations (r = 0.472; *p* = 0.014) and **b** maternal plasma and human milk ghrelin concentrations (r = −0.320; *p* = 0.017). Scatterplots are based on one imputed dataset generated by multiple imputation, whereas the correlation coefficient and *P* value are pooled across all imputed datasets. Sample size: *n* = 55.

Leptin, ghrelin, and adiponectin concentrations in maternal serum (or plasma) and human milk are presented in Table 1. The concentrations of the hormones in human milk were significantly lower than those in maternal blood (*p* < 0.001). A direct correlation was observed between maternal serum and human milk leptin concentrations (Fig. 1, r = 0.472; *p* = 0.014), whereas a significant inverse correlation was found between maternal plasma and human milk ghrelin concentration (r = −0.320; *p* = 0.017). No significant correlations were found between maternal plasma and milk adiponectin concentrations (*p* > 0.05). In addition, maternal serum concentrations of adiponectin and leptin were directly correlated (r = 0.340; *p* = 0.013).

Most of the adolescents (96.3%) had their babies at term (39.3 ± 1.5 weeks of gestation) and by vaginal delivery (~51%). All the babies were breastfed, most of them (82%) with exclusive breastfeeding. Z-score values for weight, length, and head circumference at birth were, on average, adequate, with only 15% of the babies showing one of these measures (weight, *n* = 4; height, *n* = 8) below the expected values for age. At 5 weeks postpartum, mean z-score values for weight and length were considered appropriate for age, with a low frequency of values below (16%) or above (2%) the expected range [27]. Concerning body composition at the same time point, the babies had an average body mass of 4.61 kg, fat mass of 0.97 kg, and lean mass of 3.67 kg (Table 1).

### Maternal body composition association with hormone concentrations in blood and human milk

Pre-pregnancy BMI was directly associated with serum leptin concentrations at 5 weeks postpartum (β = 1.03; 95% CI: 0.059, 1.99). The following maternal body measurements at 5 weeks were also directly associated with serum leptin at the same time: BMI (β = 1.08; 95% CI: 0.194, 1.96), total body mass (β = 0.387; 95% CI: 0.078, 0.696), total fat mass (%: β = 1.04; 95% CI: 0.479, 1.60; kg: β = 0.689; 95% CI: 0.208, 1.17), and almost all regional fat measures, especially at the arms (β = 5.95; 95% CI: 1.65, 10.3), android (β = 5.90; 95% CI: 0.871, 10.9), and gynoid region (β = 3.83; 95% CI: 1.26, 6.41) (Table 2). No significant associations were found between maternal characteristics and plasma adiponectin or ghrelin concentrations (Table 2).

**Table 2:** Associations between blood and human milk hormone concentrations and mother’s characteristics.

	Blood			Human milk		
Mother´s characteristics	Adiponectin (µg/ml)	Leptin (ng/ml)	Ghrelin (pg/ml)	Adiponectin (µg/ml)	Leptin (ng/ml)	Ghrelin (pg/ml)
	β (95% CI)	β (95% CI)	β (95% CI)	β (95% CI)	β (95% CI)	β (95% CI)
Pre-pregnancy BMI (kg/m²)	0.104 (−0.072; 0.279)	**1.03 (0.059; 1.99)**	1.09 (−26.9; 29.0)	0.003 (−0.002; 0.009)	**0.046 (0.010; 0.081)**	−0.308 (−2.63; 2.01)
Gestational weight gain (kg)	0.034 (−0.078; 0.146)	0.352 (−0.190; 0.894)	−10.7 (−27.1; 5.71)	0.001 (−0.003; 0.004)	**0.028 (0.007; 0.049)**	0.931 (−0.455; 2.32)
BMI (kg/m²)	0.076 (−0.081; 0.234)	**1.08 (0.194; 1.96)**	−8.74 (−33.5; 16.0)	0.001 (−0.003; 0.006)	**0.046 (0.014; 0.078)**	0.613 (−1.45; 2.67)
Total body mass (kg)	0.022 (−0.032; 0.075)	**0.387 (0.078; 0.696)**	−4.49 (−12.9; 3.87)	0.000 (−0.001; 0.002)	**0.017 (0.007; 0.028)**	0.134 (−0.571; 0.839)
Fat mass						
Total (%)	0.084 (−0.035; 0.202)	**1.04 (0.479; 1.60)**	−5.43 (−24.2; 13.4)	0.002 (−0.002; 0.005)	**0.048 (0.022; 0.074)**	1.20 (−0.336; 2.73)
Total (kg)	0.039 (−0.046; 0.124)	**0.689 (0.208; 1.17)**	−5.91 (−19.2; 7.39)	0.001 (−0.001; 0.004)	**0.032 (0.015; 0.049)**	0.389 (−0.722; 1.50)
Arms (kg)	0.355 (−0.453; 1.16)	**5.95 (1.65; 10.3)**	−68.5 (−194; 57.2)	0.009 (−0.016; 0.033)	**0.294 (0.132; 0.455)**	5.67 (−4.77; 16.1)
Legs (kg)	0.119 (−0.113; 0.352)	**1.90 (0.614; 3.19)**	−10.8 (−47.3; 25.7)	0.001 (−0.006; 0.008)	**0.087 (0.041; 0.132)**	1.53 (−1.49; 4.54)
Trunk (kg)	0.084 (−0.085; 0.252)	**1.30 (0.292; 2.30)**	−12.6 (−38.9; 13.7)	0.003 (−0.002; 0.008)	**0.060 (0.027; 0.093)**	0.884 (−1.32; 3.08)
Android (kg)	0.379 (−0.437; 1.20)	**5.90 (0.871; 10.9)**	−57.9 (−185; 69.6)	0.020 (−0.004; 0.045)	**0.280 (0.118; 0.443)**	4.41 (−6.18; 15.0)
Gynoid (kg)	0.228 (−0.254; 0.711)	**3.83 (1.26; 6.41)**	−11.5 (−87.4; 64.4)	0.000 (−0.014; 0.015)	**0.170 (0.069; 0.272)**	2.77 (−3.54; 9.10)
Visceral (kg)	0.091 (−2.84; 3.02)	11.9 (−5.70; 29.5)	−71.7 (−540; 397)	**0.088 (0.002; 0.173)**	0.529 (−0.077; 1.14)	4.76 (−33.1; 42.6)
Lean mass (kg)	0.031 (−0.091; 0.153)	0.574 (−0.101; 1.25)	−10.9 (−29.7; 7.95)	−0.001 (−0.004; 0.003)	**0.024 (0.000; 0.048)**	−0.103 (−1.70; 1.49)

In each model, we included hormone concentrations (adiponectin, leptin, or ghrelin) as the dependent variable, and the mother´s characteristics (BMI before pregnancy, gestational weight gain, postpartum weight retention, BMI, total body mass, fat mass, and lean mass) as the independent variables, considering postpartum period (days) as adjustment variable.

*BMI* body mass index, *β* regression coefficient, *CI* confidence interval.

Bold font indicates *P* < 0.05.

Maternal weight and several body composition measures were associated with human milk leptin concentrations (Table 2), especially fat mass at the arms (β = 0.294; 95% CI: 0.132, 0.455) and android region (β = 0.280; 95% CI: 0.118, 0.443). Visceral fat was also associated with adiponectin (β = 0.088; 95% CI: 0.002, 0.173) concentrations in human milk (Table 2).

### Human milk hormone concentrations associations with infant’s body composition

**Fig. 2:**
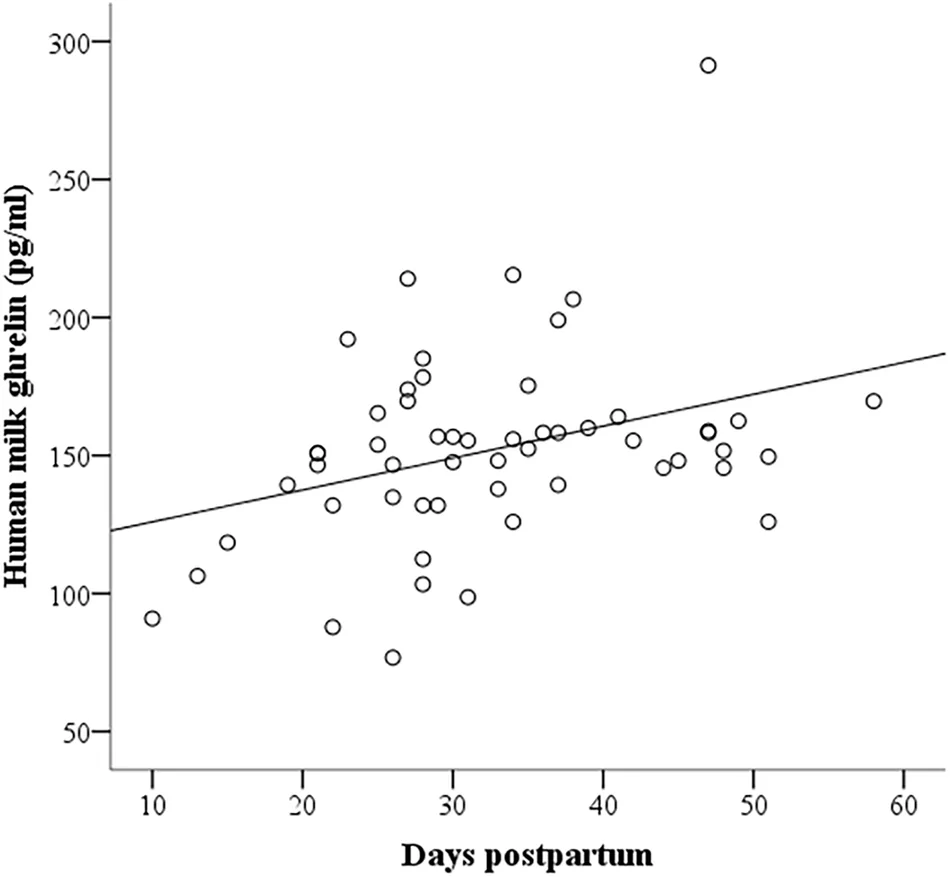
Associations between days postpartum and human milk ghrelin concentrations. The scatterplot is based on one imputed dataset generated by multiple imputation, whereas the correlation coefficient and *P* value are pooled across all imputed datasets (r = 0.354; *p* = 0.008). Sample size: *n* = 55.

Infant age was not associated with leptin and adiponectin concentrations, but was directly associated with human milk ghrelin concentrations (Fig. 2, r = 0.354; *p* = 0.008). Leptin concentration in human milk was inversely associated with infant length (β = −1.21; 95% CI: −2.24, −0.183; Table 3). Human milk ghrelin concentrations were weakly and inversely associated with weight gain since birth (β = −0.004; 95% CI: −0.007, 0.000), weight-for-age (β = −0.015; 95% CI: −0.025, −0.005), length-for-age (β = −0.008; 95% CI: −0.014, −0.002), weight-for-length (β = −0.015; 95% CI: −0.025, −0.005), BMI-for-age (β = −0.013; 95% CI: −0.021, −0.002), total body mass (β = −0.005; 95% CI: −0.009, −0.002) and lean mass (β = −0.003; 95% CI: −0.005, −0.001) (Table 3). No significant associations were found between human milk adiponectin and infant characteristics (Table 3).

**Table 3:** Associations between human milk hormone concentrations and infant’s characteristics.

Infant’s characteristics	Adiponectin (µg/ml)	Leptin (ng/ml)	Ghrelin (pg/ml)
	β (95% CI)	β (95% CI)	β (95% CI)
Weight (kg)	−0.718 (−2.20; 0.765)	−0.178 (−0.440; 0.085)	−0.004 (−0.007; 0.000)
Length (cm)	−0.099 (−5.82; 5.63)	**−1.21 (−2.24; −0.183)**	0.006 (−0.008; 0.019)
Weight gain since birth (kg)	−0.718 (−2.20; 0.765)	−0.178 (−0.440; 0.085)	**−0.004 (−0.007; 0.000)**
Length gain since birth (cm)	0.534 (−6.57; 7.64)	−0.714 (−1.99; 0.562)	−0.008 (−0.025; 0.008)
Weight-for-age (*z*-score)	−1.71 (−6.32; 2.90)	0.330 (−0.628; 1.29)	**−0.015 (−0.025; −0.005)**
Length-for-age (*z*-score)	−0.614 (−3.30; 2.07)	−0.302 (−0.794; 0.189)	**−0.008 (−0.014; −0.002)**
Weight-for-length (*z*-score)	−1.71 (−6.32; 2.90)	0.330 (−0.628; 1.29)	**−0.015 (−0.025; −0.005)**
BMI-for-age (*z*-score)	−1.16 (−4.90; 2.58)	0.022 (−0.734; 0.778)	**−0.013 (−0.021; −0.005)**
Total body mass (kg)	−1.29 (−2.91; 0.328)	0.075 (−0.222; 0.371)	**−0.005 (−0.009; −0.002)**
Total body fat (%)	−4.81 (−16.7; 7.06)	1.35 (−0.896; 3.59)	−0.025 (−0.052; 0.003)
Total body fat (kg)	−0.625 (−1.79; 0.545)	0.191 (−0.017; 0.398)	−0.002 (−0.005; 0.000)
Lean mass (kg)	−0.665 (−1.75; 0.416)	−0.116 (−0.303; 0.071)	**−0.003 (−0.005; −0.001)**

In each model, we included infant’s characteristics (weight, length, weight-for-age, length-for-age, weight-for-length, BMI-for-age, total body mass, total body fat, and lean mass) as the dependent variable, and the hormone concentrations (adiponectin, leptin, or ghrelin) as the independent variables, considering postpartum period (days) and birth weight (kg) as adjustment variables.

*BMI* body mass index, *β* regression coefficient, *CI* confidence interval.

Bold font indicates *P* < 0.05.

## Discussion

We hypothesized that maternal body composition during the first months postpartum would be associated with adiponectin, ghrelin, and leptin concentrations in maternal blood and human milk. Consistent with this hypothesis, greater maternal fat in central and appendicular regions was associated with higher leptin concentrations in serum and in human milk. In general, maternal body composition was not associated with adiponectin or ghrelin concentrations in blood or milk. We also found that higher milk leptin concentrations were associated with shorter infant length. Milk adiponectin showed no association with infant body composition, whereas higher milk ghrelin concentrations were associated with lower infant growth measures.

### Leptin

Previous studies have reported higher leptin concentrations in maternal circulation compared to human milk [8, 32, 33] as well as a direct association between them [8, 32], as also observed in the present study. Given that leptin is primarily secreted by adipose tissue and reflects maternal energy stores, higher maternal adiposity—assessed by BMI, gestational weight gain, body mass, or fat mass—is expected to result in elevated serum leptin, which in turn contributes to higher leptin concentrations in human milk, as frequently reported in the literature [6, 8, 12, 19, 32–34]. Accordingly, we found that pre-pregnancy and postpartum indicators of maternal fat accumulation, including BMI, total body mass, fat mass, and particularly arm and android fat mass, were directly associated with serum and milk leptin concentrations during the first month postpartum. These findings suggest that leptin in human milk may largely reflect maternal adiposity, although local regulatory mechanisms within the mammary gland may also contribute. This, in turn, may be relevant to early infant growth and development.

The first 6 months of life represent a critical window for adiposity programming [35]. Experimental studies have demonstrated that leptin from breast milk can be absorbed by the infant’s gastrointestinal tract and enter the circulation [36]. Adequate exposure to appropriate amounts of leptin from maternal milk appears important for normal adipose tissue development and regulation of energy homeostasis, with long-term metabolic consequences [3]. Consistently, higher human milk leptin concentrations at approximately 1 month postpartum have been associated with lower infant length, as well as lower absolute and percent total and trunk fat mass at 6 months [14]. In the present study, in which milk leptin and infant measures were assessed (~5 weeks postpartum) concurrently, milk leptin was not associated with infant weight or fat mass percentage. Nevertheless, we observed that each 0.10 ng/ml increase in milk leptin was associated with a 0.121 cm lower infant length. Although it may be of limited clinical relevance, when considered alongside existing literature [14], the findings suggest a modestly slower rate of growth among infants of mothers with higher milk leptin concentrations—typically those with higher BMI—supporting the proposed obesity-protective role of breastfeeding [6].

### Adiponectin

Higher adiponectin concentrations have consistently been reported in maternal circulation compared with human milk [32, 33], and several, but not all [4, 37], studies describe a positive association between serum and milk adiponectin [32, 38–40]. In our study, circulating adiponectin concentrations were significantly higher than those in human milk, with no association between them, supporting the hypothesis that the mammary gland acts as a local source of adiponectin synthesis, as previously suggested [4, 11, 33].

Evidence regarding the relationship between maternal adiposity and milk adiponectin remains inconsistent, with reports of direct [38, 41], inverse [17, 34], or null associations [15, 19, 42]. In the present study, maternal pre- and postpartum adiposity measures were not associated with milk adiponectin concentrations at 5 weeks postpartum, except for a weak positive association with visceral fat. This finding may reflect a compensatory mechanism in a proinflammatory maternal environment, increasing the presence of an anti-inflammatory hormone and aligning with the protective role of breastfeeding against obesity [43].

Previous studies have shown that higher milk adiponectin concentrations are associated with lower infant growth measures (weight, length, waist circumference, and BMI-for-age and weight-for-age z-scores) during the first 6 months of life [13, 16, 44] and may influence catch-up growth later in infancy [40]. However, consistent with our results, other studies have not observed such associations during early infancy, indicating that the role of milk adiponectin in early growth regulation remains unclear [4, 19].

### Ghrelin

We observed higher ghrelin concentrations in maternal circulation than in human milk, as previously reported [6, 33]. However, correlations between these compartments remain unclear, with studies reporting no association [4], weak positive association [45], or even inverse association [6], the latter also observed in the present study. This lack of consensus in the literature reflects the uncertainty about ghrelin origin in human milk [46]. In addition, we found a direct association between milk ghrelin concentrations and days postpartum, in agreement with evidence showing a progressive rise in ghrelin during lactation [45, 47]. It has been suggested that this increase is related to maternal postpartum weight loss and to the progressive increase in milk volume over the course of lactation [45, 47]. Consistent with previous reports [6, 16], milk ghrelin concentrations in the present study were not associated with maternal adiposity, suggesting that their regulation is independent of maternal body composition. Other factors may therefore be involved in regulating ghrelin concentrations in human milk, potentially including infant characteristics or nutritional demands.

The regulatory role of ghrelin in short-term food intake is well established, and there is some evidence suggesting its involvement in the long-term regulation of energy homeostasis and body weight, including in breastfeeding infants [16, 18, 48]. Higher ghrelin concentrations in milk have been reported in mothers of infants with normal weight gain during the first month compared with those showing excessive weight gain [18]. Similarly, mean human milk ghrelin concentrations were higher in mothers of normal-weight infants than in mothers of obese infants [16]. In the present study, we also observed weak inverse associations between milk ghrelin concentrations and several infant growth measures, including weight gain since birth. Although these associations were modest, they are consistent with the hypothesis that ghrelin in human milk may be physiologically regulated in response to the infant’s nutritional status and growth needs, potentially contributing to postnatal growth regulation [4]. However, bidirectional influences between infant growth and human milk ghrelin cannot be excluded.

A limitation of our study is the use of homogenized whole milk instead of skim milk for hormone analysis, as lipids may interfere with certain assays, particularly for leptin and adiponectin [1]. Nevertheless, the measurement of these hormones in whole milk using ELISA assays has been established and validated, as it better represents the milk consumed by the infant [49]. Furthermore, human milk samples consisted of 20 mL collected from the non-suckled breast immediately after the last feeding, rather than fully expressed samples, which may have affected metabolic hormone concentrations [48, 49]. In addition, we are aware that a single morning milk sample is subject to circadian fluctuations [47, 50]. To minimize this variability, the timing of collection was standardized as described in the methods section. Importantly, we did not have access to estimates of the total volume of human milk ingested by the infant, which may have influenced the interpretation of hormone exposure. Our sample consisted exclusively of adolescent mothers, and the specific physiological adaptations of this period may influence metabolic and hormonal regulation, although the effect of maternal age on human milk composition remains inconclusive [50, 51], thereby limiting the generalizability of these findings. The strength of this study lies in the use of DXA to simultaneously assess the body composition of mother–infant pairs, providing more accurate estimates, in addition to the inclusion of widely used child growth indicators [27].

In these predominantly eutrophic adolescent mothers, the findings suggest that maternal adiposity contributes to leptin concentrations in both serum and human milk. In contrast, results on blood and milk adiponectin and ghrelin suggest a regulation mechanism less dependent on maternal fat mass. Although the findings should be interpreted with caution, given the cross-sectional design, our results indicate that leptin and ghrelin in human milk may be involved in infant growth and body composition, whereas adiponectin does not appear to play a major role. Taken together, the results indicate that the relationships between maternal body composition, milk hormone concentrations, and infant growth are hormone-specific and do not reflect a uniform biological process. Further longitudinal studies are needed to clarify these associations and their potential implications for early growth and development.

## Data Availability

The data supporting the findings of this study are available from the corresponding author upon reasonable request.
